# Detection of Sars-Cov-2 antigens in thyroid gland showing histopathological features of subacute thyroiditis

**DOI:** 10.1530/ETJ-22-0005

**Published:** 2022-02-15

**Authors:** Hrvoje Jakovac, Antun Ferenčić, Christophe Stemberger, Bojana Mohar Vitezić, Dražen Cuculić

**Affiliations:** 1 Department of Physiology, Immunology and Pathophysiology, Faculty of Medicine, University of Rijeka, Rijeka, Croatia; 2 Department of Forensic Medicine and Criminalistics, Faculty of Medicine, University of Rijeka, Rijeka, Croatia; 3 Department of Pathology, Clinical Hospital Centre Rijeka, Rijeka, Croatia; 4 Department of Microbiology and Parasitology, Faculty of Medicine, University of Rijeka, Rijeka, Croatia

**Keywords:** Sars-Cov-2, COVID-19, spike protein, thyroid gland, subacute thyroiditis

## Abstract

The clinical and laboratory findings of subacute thyroiditis have been repeatedly reported as being associated with acute Sars-Cov-2 infection and post-COVID-19 syndrome. The exact mechanisms and histopathological correlations underlying thyroid involvement remained unresolved, but current insights suggest either direct viral damage, systemic inflammatory reaction, or an autoimmune response as possible noxious effectors. Here we present findings of immunohistochemical/immunofluorescence detection of Sars-Cov-2 viral proteins (spike/S and nucleocapside proteins) in relation to histoarchitectonic changes of autoptic thyroid tissue obtained from patient who deceased from COVID-19.

## Images in thyroidology

Clinical and laboratory findings of subacute thyroiditis have been repeatedly reported as being associated with acute Sars-Cov-2 infection and post-COVID-19 syndrome (1, 2, 3). Exact mechanisms and histopathological correlates underlying thyroid involvement remained unresolved, but current insights suggest either direct viral damage, systemic inflammatory reaction or an autoimmune response as possible noxious effectors (4).

Here, we present findings of immunohistochemical/immunofluorescence detection of Sars-Cov-2 viral proteins in relation to histoarchitectonic changes of post-mortem thyroid tissue obtained from the patient who deceased from COVID-19.

**Figure 1:**
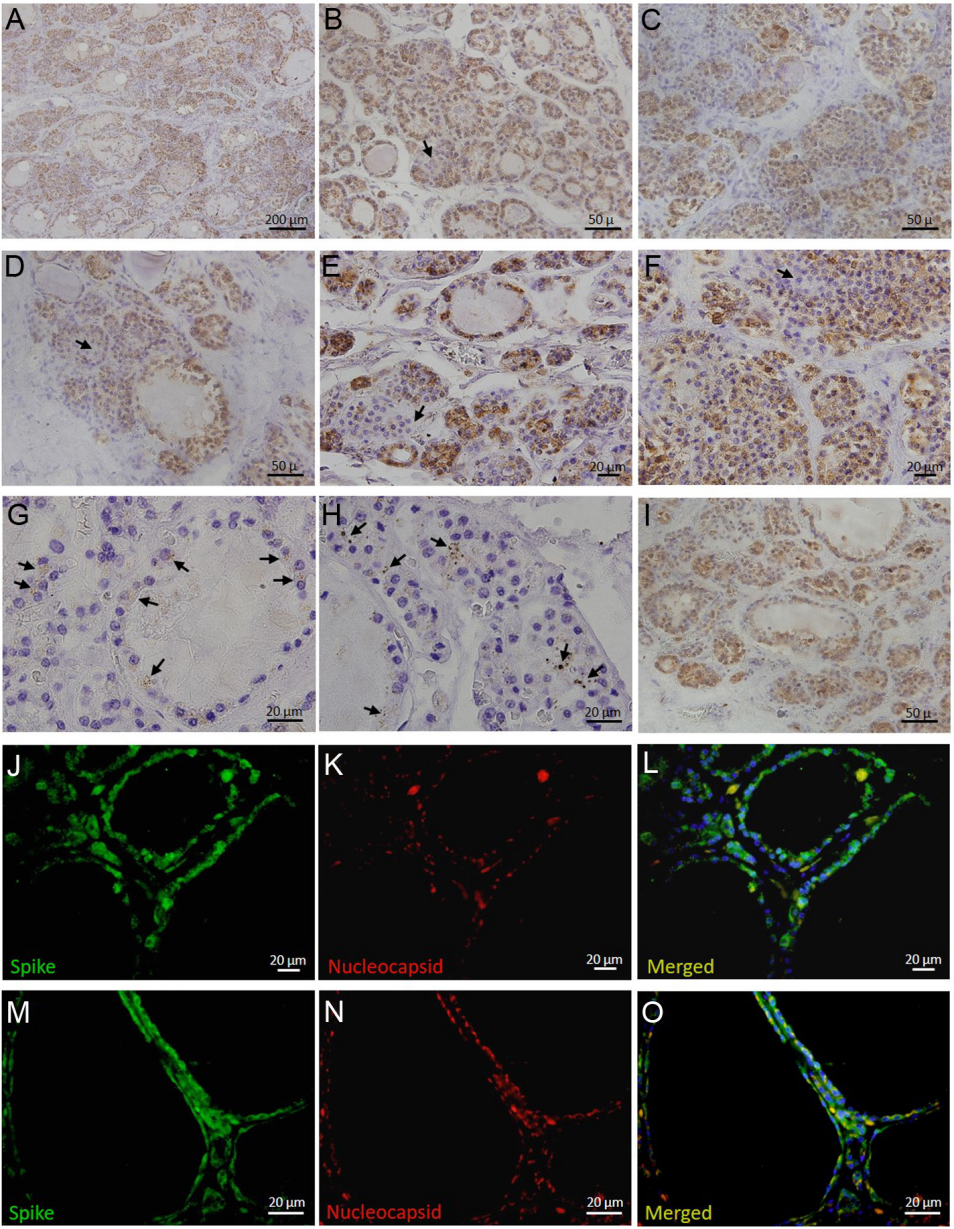
Immunohistochemical/immunofluorescence expression of Sars-Cov-2 antigens in relation to histopathological changes in thyroid gland obtained from patient who deceased from COVID-19. Sars-Cov-2 spike (S) protein was found notably expressed throughout the thyroid tissue and was localized diffusely in the cytoplasm of follicular cells (A, B, C, D, E, F and J, K, L, M, N, O). Concomitantly, the thyroid gland harboured copious mononuclear infiltrate (A, B, C, D, E, F, G and H) forming granuloma-like structures (B, C, D, E and F) with giant multinuclear cells (arrows on B, D, E, F), epithelial desquamation, colloid depletion and extensive follicular disruption (A, B, C, D, E, F, G, H and I, corresponding to findings consistent with subacute thyroiditis. Spike protein positivity was also found in epithelioid cells within the granulomatous tissue (F). Sars-Cov-2 nucleocapsid protein immunopositivity was noticeably sparser, arranged in punctiform inclusions and localized predominantly in the perinuclear region of follicular cells (arrows on G and H; J, K, L, M, N, O). The majority of remaining follicular cells showed positivity for cleaved caspase-3 (I). Staining was performed using rabbit anti-SARS-CoV-2 spike glycoprotein antibody (Abcam, ab272504; dilution 1:4000), mouse anti-SARS-CoV-2 nucleocapsid protein antibody (Cell Signaling Technology, clone E8R1L; dilution 1:200) and rabbit anti-caspase-3 antibody (Abcam, ab13847; diluted 1:50). Immunoreactions were visualized by DAKO EnVision+System (DAKO Cytomation) for immunohistochemistry or by Alexa Fluor 488 donkey anti-rabbit IgG (Thermo Fisher Scientific; diluted 1:300) and Alexa Fluor 555 goat anti-mouse IgG (Thermo Fisher Scientific; diluted 1:500) secondary antibodies for immunofluorescence studies. Magnifications: A × 100; B, C, D, E, I × 400; F, J, K, L × 600; G, H, M, N, O × 1000.

Having been found unconscious in his home, the 85-year-old man was transported to the emergency department. He was severely tachypneic (50/min), tachycardic (160/min), hypotensive (50/30 mmHg) and cyanotic, with immeasurable SpO_2_ by pulse oximeter. The patient was immediately treated with high oxygen flow therapy (15 L/min), noradrenaline (5 mg/50 mL) and volume substitution (plasmalyte). Rapid antigen test and RT-PCR showed Sars-Cov-2 positivity. There were no vaccination data in his records. Arterial blood gas analysis, taken when the patient was on oxygen therapy, revealed pH of 7.30, pCO_2_ of 4.1 kPa, pO_2_ of 4.2 kPa and sO_2_ of 51.7%. Other laboratory findings were significant for elevated CRP (59.6 mg/L) and leukopenia (3.0 × 10^9^/L). The patient's condition was fulminantly deteriorating, and mechanical ventilation was introduced, but despite intensive treatment, he died 4 h after admission. The body underwent autopsy which showed acute respiratory distress syndrome as a cause of death. Besides, samples of thyroid gland obtained from the deceased patient were found to express striking Sars-Cov-2 spike glycoprotein immunopositivity throughout the tissue, localized diffusely in cytoplasm of follicular cells (Fig. 1A, B, C, D, E, F and J, K, L, M, N, O). This finding was associated with the presence of copious mononuclear infiltrate forming granuloma-like structures with giant multinucleated cells, as well with extensive follicular disruption/collapse along with epithelial desquamation and colloid depletion (Fig. 1A, B, C, D, E, F, G, H and I), being consistent with histopathological characteristics of subacute thyroiditis. Spike protein positivity was also found in epithelioid cells within the granuloma fulfilling destroyed follicles (Fig. 1F). In contrast, nucleocapsid protein immunopositivity was noticeably sparser, forming punctiform clusters with predominant perinuclear localization in thyrocytes (Fig. 1, arrows on G and H; J, K, L, M, N, O). Such expression discordance between these Sars-Cov-2 antigens, similar having also been demonstrated in other epithelial tissues (5), suggests the possibility of S protein shedding in the thyroid gland, leaving behind targets of the immune system even after viral clearance. Interestingly, the vast majority of remnant thyrocytes were immunopositive for cleaved caspase-3 (Fig. 1I), pointing to apoptosis as an important mechanism in SARS-CoV-2-induced thyroid pathology.

## Declaration of interest

The authors declare that there is no conflict of interest that could be perceived as prejudicing the impartiality of this case report.

## Funding

This work was supported by the University of Rijeka (grant number uniri-biomed-18-187).

## Statement of ethics

Research was conducted according to the guidelines of the Declaration of Helsinki. The deceased patient had no family members who could have given informed consent. Study was approved by the Ethics Committee of Medical Faculty, University of Rijeka.

## Author contribution statement

H J performed immunohistochemical and immunofluorescent staining, analyzed images, reviewed the literature, drafted and wrote the manuscript. A F, C S and D C performed autopsy and pathological examinations. B M V performed RT-PCR analysis. All authors read and approved the final manuscript.
